# Association of glucagon-like peptide-1 receptor agonists with atrial fibrillation recurrence after catheter ablation: a systematic review and meta-analysis

**DOI:** 10.3389/fcvm.2026.1868320

**Published:** 2026-08-27

**Authors:** Dan He, Defu Lv, Jiaxin Tang, Xiaojuan Liao, Ping Mao, Zhu Tian

**Affiliations:** 1 Department of Emergency Medicine, Shapingba Hospital Affiliated to Chongqing University (Shapingba District People’s Hospital of Chongqing), Chongqing, China; 2 Department of Emergency Medicine, Chongqing Hospital, The First Affiliated Hospital of Guangzhou University of Chinese Medicine (Chongqing Beibei District Hospital of Traditional Chinese Medicine), Chongqing, China

**Keywords:** atrial fibrillation recurrence, catheter ablation, glucagon-like peptide-1 receptor agonists, meta-analysis, systematic review

## Abstract

**Background:**

Current studies suggest a potential association between the use of glucagon-like peptide-1 receptor agonists (GLP-1RAs) and atrial fibrillation (AF) recurrence after catheter ablation, but the findings remain controversial. Therefore, we conducted a meta-analysis to evaluate this association, aiming to provide evidence for clinical prevention and treatment.

**Methods:**

We systematically searched PubMed, Embase, Web of Science, Cochrane Library, and four Chinese databases (CNKI, WanFang, VIP, and CBM) up to June 10, 2026, for studies on the association between GLP-1RAs and AF recurrence following catheter ablation. Two researchers independently screened literature, extracted data and assessed study quality. Statistical analyses were performed using Review Manager 5.3 and R 4.6.0.

**Results:**

A total of seven studies involving 7,907 patients were included. The meta-analysis showed that the use of GLP-1RAs was associated with a reduced risk of AF recurrence after catheter ablation (HR = 0.52, 95%CI = 0.37–0.73, *P* < 0.001). Subgroup analyses consistently demonstrated a protective effect of GLP-1RAs on AF recurrence, irrespective of sample size (≤200: HR = 0.34, 95%CI = 0.22–0.52, *P* < 0.001 vs. > 200: HR = 0.70, 95%CI = 0.53–0.91, *P* = 0.009), study design (prospective: HR = 0.56, 95%CI = 0.43–0.72, *P* < 0.001 vs. retrospective: HR = 0.47, 95%CI = 0.25–0.89, *P* = 0.020), or follow-up duration (≤12 months: HR = 0.53, 95%CI = 0.40–0.72, *P* < 0.001 vs. > 12 months: HR = 0.58, 95%CI = 0.35–0.95, *P* *=* 0.030).

**Conclusions:**

The use of GLP-1RAs may reduce the risk of AF recurrence after catheter ablation. However, the 95% prediction interval crossed the null value, indicating that this protective effect is not definitive. Given the limitations of this study, further high-quality randomized controlled trials are warranted to confirm these findings.

## 1 Introduction

Atrial fibrillation (AF) is the most prevalent sustained arrhythmia in clinical practice (1). In 2019, there were 59.7 million patients with AF worldwide, approximately twice the number recorded in 1990 (2). With the rapid aging of modern society, the incidence and prevalence of AF are gradually increasing (3, 4). Stroke, thromboembolism and heart failure are common complications in patients with AF, significantly impairing their quality of life and placing a heavy burden on society and healthcare systems (5, 6).

Catheter ablation, as an effective rhythm control strategy for AF, can significantly reduce the onset of AF, improve quality of life, and delay disease progression (7, 8). However, postoperative recurrence remains the primary clinical challenge of catheter ablation for AF. Studies have shown that the recurrence rate of AF within the first year following ablation ranges from 30% to 50% (9–11), and this is even more evident in patients with obesity, type 2 diabetes, and hypertension (12–14). Therefore, it is of great clinical significance to identify and intervene modifiable risk factors for AF recurrence.

Glucagon-like peptide-1 receptor agonists (GLP-1RAs) are a new class of hypoglycemic drugs (15, 16) that promote insulin secretion, inhibit glucagon release, and delay gastric emptying by activating GLP-1 receptor. They also confer multiple cardiovascular benefits, including weight loss, blood pressure regulation, and lipid reduction (17–20). GLP-1 receptors are not only expressed in pancreatic *β*-cells but also widely distributed in cardiomyocytes, vascular endothelial cells, smooth muscle cells, and cardiac fibroblasts (21), suggesting that GLP-1RAs may have direct or indirect effects on the cardiovascular system. Previous studies have shown that the cardioprotective effects of GLP-1RAs are mediated through multiple mechanisms, including anti-inflammatory and anti-oxidative stress pathways, regulation of myocardial metabolism, and delay of atrial fibrosis (22–24).

GLP-1RAs have received substantial attention over the past decade due to their significant cardiovascular benefits observed in diabetic patients (25). However, the potential association between GLP-1RAs and AF recurrence after catheter ablation remains controversial. While multiple studies have observed that the use of GLP-1RAs significantly reduces the risk of AF recurrence after catheter ablation (26, 27), others have not found a statistically significant association (28, 29). To address this controversy, we conducted this meta-analysis to evaluate the association between GLP-1RAs and AF recurrence after catheter ablation, aiming to provide evidence for clinical prevention and treatment.

## 2 Method

This review was conducted in accordance with the recommendations of the Preferred Reporting Items for Systematic Reviews and Meta-Analyses (PRISMA) statement (30).

### 2.1 Search strategy

We systematically retrieved literature from PubMed, Embase, Web of Science and Cochrane Library up to June 10, 2026, on the association between GLP-1RAs and AF recurrence following catheter ablation. Additionally, to minimize language bias, we also searched four Chinese biomedical databases: China National Knowledge Infrastructure (CNKI), WanFang database, Chinese Scientific Journal Database (VIP) and China Biology Medicine disc (CBM). The search strategy combined Medical Subject Headings (MeSH) and free words. The search terms included the following: glucagon-like peptide-1 receptor agonists, liraglutide, semaglutide, tirzepatide, dulaglutide, exenatide, albiglutide, lixisenatide, atrial fibrillation, etc. The complete search strategy is provided in the Supplementary Material (Supplementary Table S1).

### 2.2 Eligibility criteria

#### 2.2.1 Inclusion criteria

(1) Participant: patients diagnosed with atrial fibrillation;
(2) Intervention: use of GLP-1RAs;
(3) Comparison: non-use of GLP-1RAs;
(4) Outcomes: AF recurrence after catheter ablation;
(5) Study design: Randomized controlled trials (RCTs) or cohort studies.

#### 2.2.2 Exclusion criteria

(1) Reviews, commentaries, case reports, case-control studies, or conference abstracts;
(2) Full text could not be retrieved;
(3) Duplicate publications.

### 2.3 Data extraction

Two researchers independently screened the literature and extracted data. The extracted information included the first author, publication year, region, data source, study design, sample size, diagnostic criteria for AF recurrence, intervention plan, follow-up duration, effect size and 95% confidence interval (CI) for the corresponding outcomes. Any disagreements were resolved by discussion or by consulting a third researcher.

### 2.4 Study quality assessment

The Risk of Bias in Non-randomized Studies-of Interventions (ROBINS-I) tool was used to assess the quality of eligible cohort studies (31). The ROBINS-I tool evaluates studies across seven domains: confounding, selection of participants, classification of interventions, deviations from intended interventions, missing data, measurement of outcomes, and selection of reported results. If all domains are judged as “low risk”, the overall risk of bias is low. If some domains are judged as “moderate risk” and no domain is judged as “serious risk” or “critical risk”, the overall risk of bias is moderate. If any domain is judged as “serious risk” and no domain is judged as “critical risk”, the overall risk of bias is serious. If any domain is judged as “critical risk”, the overall risk of bias is critical. The Cochrane Risk of Bias tool version 2.0 (RoB 2.0) was used to assess the risk of bias in RCTs (32). RoB 2.0 evaluates risk across five domains: the randomization process, deviations from intended interventions, missing outcome data, measurement of the outcome, and selection of the reported result. If all domains are judged as “low risk”, the overall risk of bias is low. If some domains are judged as “some concerns” and no domain is judged as “high risk”, the overall risk of bias is some concerns. If any domain is judged as “high risk”, the overall risk of bias is high. Two researchers independently performed the assessments, and any disagreements were resolved through discussion or by consulting a third researcher for adjudication.

### 2.5 Data synthesis and analysis

Statistical analyses were performed using Review Manager 5.3 and R 4.6.0. Since the results of Zou et al. and Salerno et al. were reported as proportions without time-to-event data (33, 34), we converted the binary data into hazard ratios (HRs) for inclusion in the meta-analysis based on the method described by Watkins et al. (35). The conversion formula was HR = ln(p_G)/ln(p_C), where *p* is the proportion free from AF recurrence. Detailed conversion calculations are provided in the Supplementary Materials (Supplementary Tables S2a, S2b).

The heterogeneity among the included studies was assessed using the *I*^2^ statistic. If *P* > 0.100 and *I*^2^ < 50%, no significant statistical heterogeneity was considered present, and a fixed-effects model was applied. Conversely, if *P* < 0.100 and *I*^2^ ≥ 50%, significant heterogeneity was considered present, and a random-effects model was used (36, 37). Subgroup analyses were performed based on sample size (≤200 vs. >200), study design (prospective vs. retrospective) and follow-up duration (≤12 months vs. >12 months) to explore potential sources of heterogeneity. Egger's test, Begg's test, funnel plot and the Trim-and-Fill method were used to assess the publication bias among the included studies. Sensitivity analysis was performed to evaluate the robustness and stability of the pooled results.

### 2.6 Certainty of evidence assessment

The certainty of evidence for the primary outcome was assessed using the Grading of Recommendations Assessment, Development and Evaluation (GRADE) framework (38). Evidence from randomized controlled trials started at high quality, while evidence from observational studies started at low quality. The certainty of evidence could be downgraded by one or more levels based on five factors: risk of bias, inconsistency, indirectness, imprecision, and publication bias.

## 3 Results

### 3.1 Study characteristics

**Figure 1:**
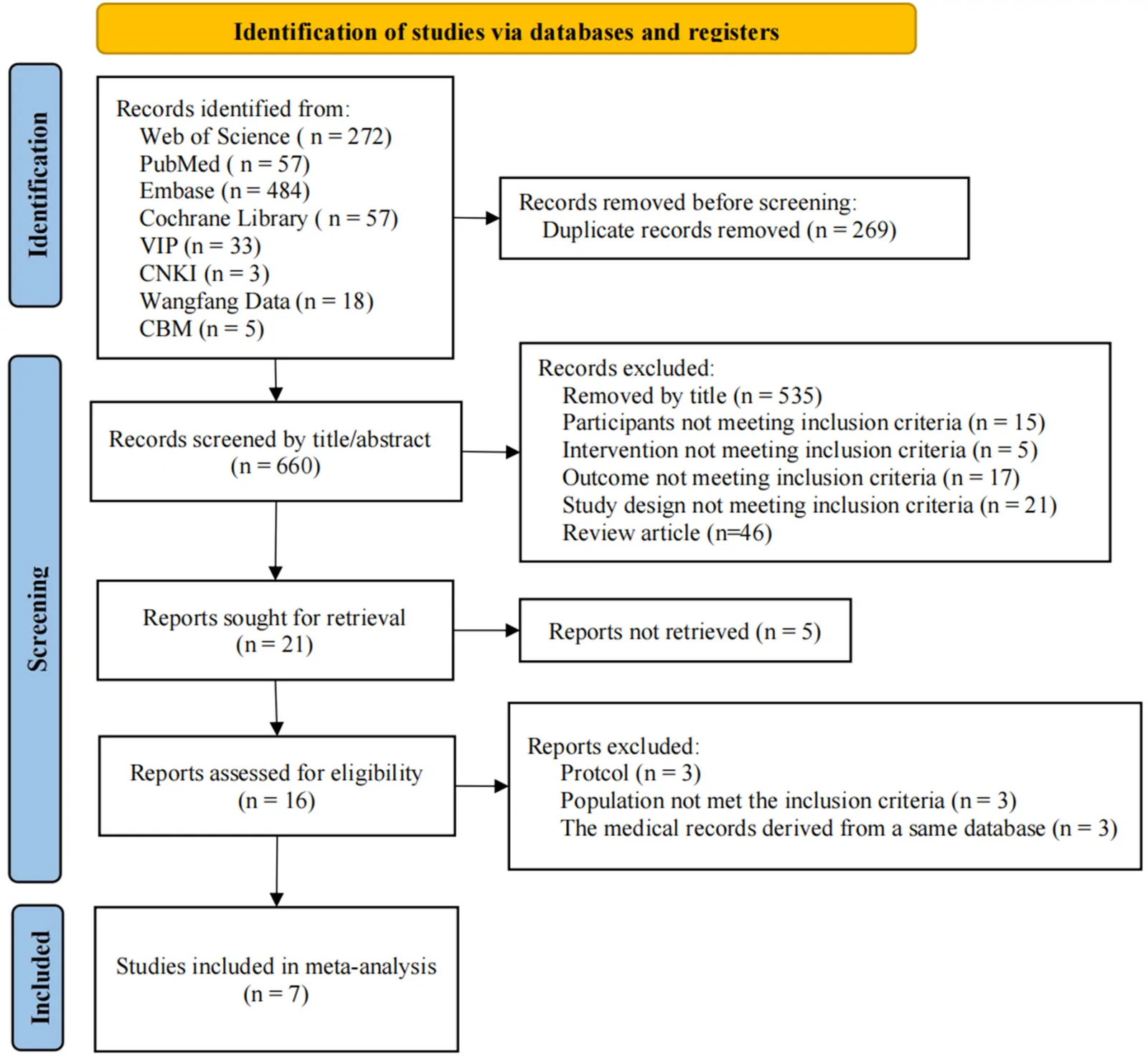
PRISMA flow diagram of studies included in this meta-analysis.

The initial database search yielded 929 studies. After removing 269 duplicate records, 660 records remained and were screened based on titles and abstracts. The remaining 21 studies were independently read by two researchers, and ultimately seven studies were included (27, 33, 34, 39–42) (Figure 1).

The general characteristics of the included studies are shown in Table 1. A total of seven studies, published between 2024 and 2026, were included, comprising one RCT and six cohort studies. In total, 7,907 participants were enrolled. Sample sizes ranged from 59 to 6,700 participants, and follow-up durations varied from 12 to 72 months. Four studies had a sample size of fewer than 200 cases, while three had more than 200 cases.

**Table 1:** General features of the studies.

Study	Design	Data source	Definition of AF recurrence	Intervention/control	Type of GLP-1RAs	Participants (No.)	Type of participants	Enrollment period (year)	Follow-up duration (months)
Tabaja et al. (41)	Retrospective study	A prospective AF ablation registry	Not reported	GLP-1RAs (not specified)/non-GLP-1RAs	Not specified	60/60	Patients undergone AF ablation with BMI >30 kg/m^2^	2018–2022	12
Zou et al. (33)	Retrospective study	Not reported	Not reported	Semaglutide/non-Semaglutide	Semaglutide	61/62	Patients undergone AF ablation	Not reported	12
Guo et al. (39)	Prospective Study	A prospective AF ablation registry	Occurrence of atrial arrhythmias lasting ≥30 s, including AF, atrial flutter, or atrial tachycardia	Semaglutide/non-Semaglutide	Semaglutide	158/279	Patients undergone AF ablation with BMI >24 kg/m^2^ and T2DM	2022–2024	12
Ciconte et al. (42)	Prospective Study	A prospective AF ablation registry	Not reported	Semaglutide/non-Semaglutide	Semaglutide	181/181	Patients undergone AF ablation BMI >30 kg/m^2^	2019–2024	18
Venier et al. (27)	Retrospective study	TriNetX research database	Hospitalization or Outpatient visit coded for AF	GLP-1RAs (multiple types)/non- GLP-1RAs	Semaglutide Liraglutide, Dulaglutide, Exenatide, Lixisenatide, Tirzepatide	3,350/3,350	Patients undergone AF ablation BMI >30 kg/m^2^	2015–2025	72
Goldberger et al. (40)	RCT	University of Miami	Not mentioned	Liraglutide/non- Liraglutide	Liraglutide	28/31	patients undergone AF ablation ≥27 kg/m^2^	2019–2026	36
Salerno et al. (34)	Retrospective study	A tertiary center in Italy	Any atrial tachyarrhythmia recurrence after a 3-month blanking period	Semaglutide/non-Semaglutide	Semaglutide	53/53	Patients undergone AF ablation	2020–2025	12

GLP-1 RAs, glucagon-like peptide-1 receptor agonists; AF, atrial fibrillation; BMI, body mass index; DM, diabetes mellitus; T2DM, type 2 diabetes mellitus; No, number; RCT, randomized controlled trial.

**Figure 2:**
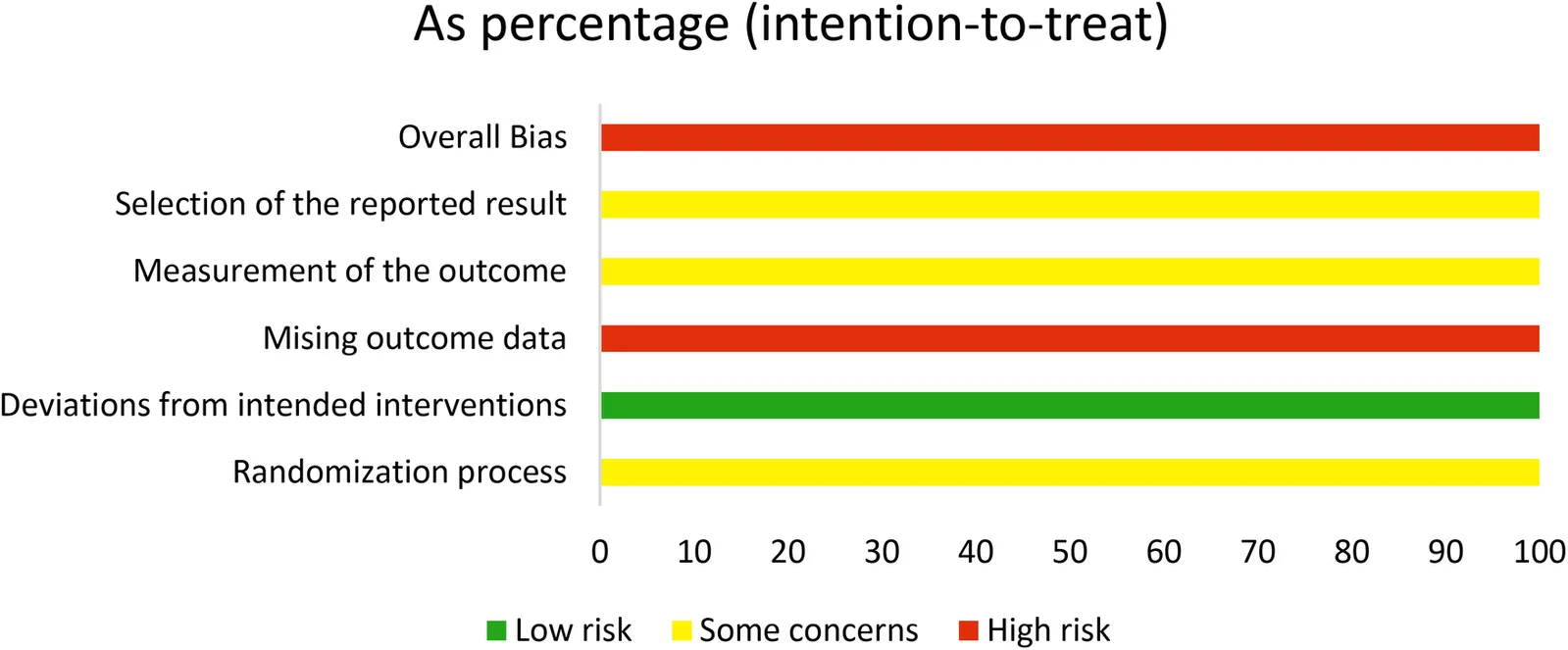
Risk of bias assessment for the single included randomized controlled trial (RCT) using Cochrane RoB 2.0 tool.

The ROBINS-I assessment for the cohort studies showed that four studies were rated as moderate risk of bias, while two studies were rated as serious risk of bias (Table 2). The overall risk of bias for the included RCT was judged as “high risk”, with one domain rated as high risk of bias (Figure 2).

**Table 2:** ROBINS-I assessment for included cohort studies.

Study	Confounding	Selection of participants	Classification of interventions	Deviations from intended interventions	Missing data	Measurement of outcomes	Selection of Reported results	Overall
Tabaja et al. (41)	Moderate	Low	Moderate	Low	Low	Moderate	Low	Moderate
Zou et al. (33)	Moderate	Moderate	Moderate	Low	Moderate	Moderate	Low	Moderate
Venier et al. 2026	Moderate	Low	Low	Low	Low	Moderate	Low	Moderate
Ciconte et al. (42)	Moderate	Low	Low	Low	Low	Low	Low	Moderate
Guo et al. (39)	Serious	Moderate	Low	Moderate	Moderate	Moderate	Low	Serious
Salerno et al. (34)	Serious	Low	Low	Low	Low	Low	Low	Serious

ROBINS-I, risk of bias in non-randomized studies—of interventions.

### 3.2 Meta-analysis results

**Figure 3:**
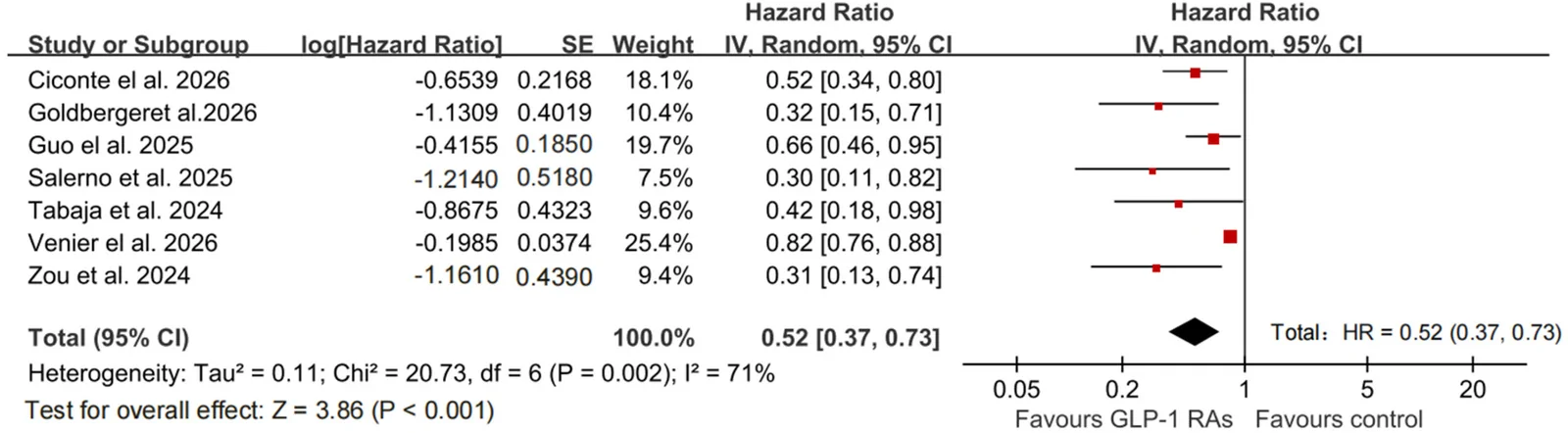
Forest plot of AF recurrence following catheter ablation for GLP-1 RAs and controls.

A total of seven studies involving 7,907 participants were included. The heterogeneity test showed *I*^2^ = 71%, *P* = 0.002; therefore, a random-effects model was adopted. The meta-analysis demonstrated that the use of GLP-1RAs significantly reduced the risk of AF recurrence after catheter ablation compared with the control group (HR = 0.52, 95%CI = 0.37–0.73, *P* < 0.001) (Figure 3). The numerical weights for each study are provided in the Supplementary Materials (Supplementary Table S3). To better account for between-study variability, we calculated the 95% prediction interval (PI), which was 0.24–1.09. Since this interval crossed 1, it suggests that the true effect of a future similar study might fall within the null range.

### 3.3 Cumulative meta-analysis results

To assess the trend of effect sizes over publication time and sample size, we performed cumulative meta-analyses stratified by publication year and by sample size.

(1) By publication year: Studies were sequentially added by year (2024–2026). The cumulative HR remained stable between 0.45 and 0.51, with the 95% confidence interval never crossing 1 (Table 3). The final pooled HR was 0.52 (95% CI = 0.37–0.73). This indicates that the conclusion of this study is not influenced by the order of publication time, and there is no bias driven by early studies.
(2) By sample size: Studies were sequentially added from the smallest to the largest sample size. The cumulative HR decreased from 0.27 (95% CI = 0.12–0.59) in the smallest-sample analysis to 0.52 (95% CI = 0.37–0.73) in the final pooled analysis, with the 95% confidence interval consistently not crossing 1 throughout the cumulative process (Table 3). This suggests that although smaller studies amplified the effect size, the direction of the conclusion remained unchanged.

**Table 3:** Cumulative meta-analysis results.

Order	Study added	Cumulative HR (95% CI)
By publication year (2024 to 2026)		
1	Tabaja et al. (41)	0.42 (0.18–0.97)
2	Zou et al. (33)	0.36 (0.20–0.66)
3	Guo et al. (39)	0.51 (0.32–0.81)
4	Salerno et al. (34)	0.46 (0.29–0.73)
5	Ciconte et al. (42)	0.51 (0.39–0.68)
6	Goldberger et al. (40)	0.45 (0.33–0.63)
7	Venier et al. (27)	0.52 (0.37–0.73)
By sample size (smallest to largest)		
1	Goldberger et al. (40)	0.27 (0.12–0.59)
2	Zou et al. (33)	0.29 (0.16–0.52)
3	Salerno et al. (34)	0.29 (0.18–0.48)
4	Tabaja et al. (41)	0.32 (0.21–0.49)
5	Ciconte et al. (42)	0.40 (0.29–0.56)
6	Guo et al. (39)	0.45 (0.33–0.63)
7	Venier et al. (27)	0.52 (0.37–0.73)

HR, hazard ratio.

### 3.4 Subgroup analysis

To explore potential sources of heterogeneity, we conducted subgroup analyses based on sample size (≤200 vs. >200), study design (prospective vs. retrospective), and follow-up duration (≤12 months vs. >12 months). The results are summarized in Table 4.

**Table 4:** Subgroup analysis.

Subgroup analysis	The number of included studies	Heterogeneity		Model	Meta analysis		*P* for interaction
		*P*-value	*I* ^2^		HR (95% CI)	*P*-value	
Sample size							0.010
≤200	4	0.950	0%	Fixed	**0.34** (**0.22, 0.52)**	**<0**.**001**	
>200	3	0.007	63%	Random	**0.70** (**0.53, 0.91)**	**0**.**009**	
Study design							0.700
Prospective study	3	0.250	28%	Fixed	**0.56** (**0.43, 0.72)**	**<0**.**001**	
Retrospective study	4	0.010	72%	Random	**0.47** (**0.25, 0.89)**	**0**.**020**	
Follow-up duration							0.550
≤12 months	4	0.220	32%	Fixed	**0.53** (**0.40, 0.72)**	**<0**.**001**	
>12 months	3	0.009	79%	Random	**0.58** (**0.35, 0.95)**	**0**.**030**	

HR, hazard ratio; CI, confidence interval.

Bold values indicate the primary meta-analysis results, including the pooled hazard ratio (HR) with its 95% confidence interval (CI) and the corresponding P-value for each subgroup.

#### 3.4.1 Subgroup analysis by sample size

Sample size ≤200: Pooled analysis of four studies showed that the use of GLP-1RAs significantly reduced the risk of AF recurrence after catheter ablation (HR = 0.34, 95% CI = 0.22–0.52, *P* < 0.001) (Table 4).

Sample size >200: Pooled analysis of three studies showed that the use of GLP-1RAs significantly reduced the risk of AF recurrence after catheter ablation (HR = 0.70, 95% CI = 0.53–0.91, *P* = 0.009) (Table 4).

The interaction test showed a significant difference between subgroups (*P* for interaction = 0.010) (Table 4).

#### 3.4.2 Subgroup analysis by study design

Prospective studies: Pooled analysis of three studies showed that the use of GLP-1RAs significantly reduced the risk of AF recurrence after catheter ablation (HR = 0.56, 95% CI = 0.43–0.72, *P* < 0.001) (Table 4).

Retrospective studies: Pooled analysis of four studies showed that the use of GLP-1RAs significantly reduced the risk of AF recurrence after catheter ablation (HR = 0.47, 95% CI = 0.25–0.89, *P* = 0.020) (Table 4).

No significant interaction was observed between subgroups (*P* for interaction = 0.700) (Table 4).

#### 3.4.3 Subgroup analysis by follow-up duration

Follow-up duration ≤12 months: Pooled analysis of four studies showed that the use of GLP-1RAs significantly reduced the risk of AF recurrence after catheter ablation (HR = 0.53, 95% CI = 0.40–0.72, *P* < 0.001) (Table 4).

Follow-up duration >12 months: Pooled analysis of three studies showed that the use of GLP-1RAs significantly reduced the risk of AF recurrence after catheter ablation (HR = 0.58, 95% CI = 0.35–0.95, *P* = 0.030) (Table 4).

No significant interaction was observed between subgroups (*P* for interaction = 0.550) (Table 4).

### 3.5 Sensitivity analysis

A leave-one-out sensitivity analysis was performed. The pooled effect estimates did not reverse direction upon the exclusion of any single study. After excluding the study by Venier et al., the heterogeneity among the remaining studies was significantly reduced (*I*^2^ = 13%, *P* = 0.330) (27). This may be because the data for that study were derived from the TriNetX database, which differs from conventional clinical case registries in terms of case selection, data recording, and outcome assessment, leading to substantial methodological heterogeneity.

Furthermore, we conducted a stratified subgroup sensitivity analysis based on the original effect measure type to assess the potential impact of the Watkins conversion method (35) on the direction of the conclusions. Two studies reporting only binary outcome data (33, 34) were assigned to the RR subgroup, while the remaining five studies (27, 39–42) reporting HRs were included in the HR subgroup. The results showed that the pooled effect size in the HR subgroup was 0.60 (95% CI = 0.44–0.82), and that in the RR subgroup was 0.35 (95% CI = 0.19–0.64) (Supplementary Tables S4a, S4b). Both subgroups demonstrated consistent effect directions, supporting the protective role of GLP-1RAs against AF recurrence, which closely aligns with the findings of the main analysis. These findings indicate that the Watkins conversion method did not materially affect the direction of our conclusions, supporting the robustness of the primary results.

Additionally, after excluding the high-risk RCT by Goldberger et al., the pooled effect size became 0.56 (95% CI = 0.41–0.78), and the direction of the effect remained unchanged, indicating that our conclusions are robust (40). Furthermore, a sensitivity analysis using robust variance estimation (RVE) yielded an adjusted HR of 0.51 (95% CI = 0.36–0.72, *P* = 0.012), consistent with the primary analysis.

Together, these sensitivity analyses confirm that our findings are not driven by data conversion methods, model assumptions, small-study bias, or any single dominant study.

### 3.6 Publication bias

**Figure 4:**
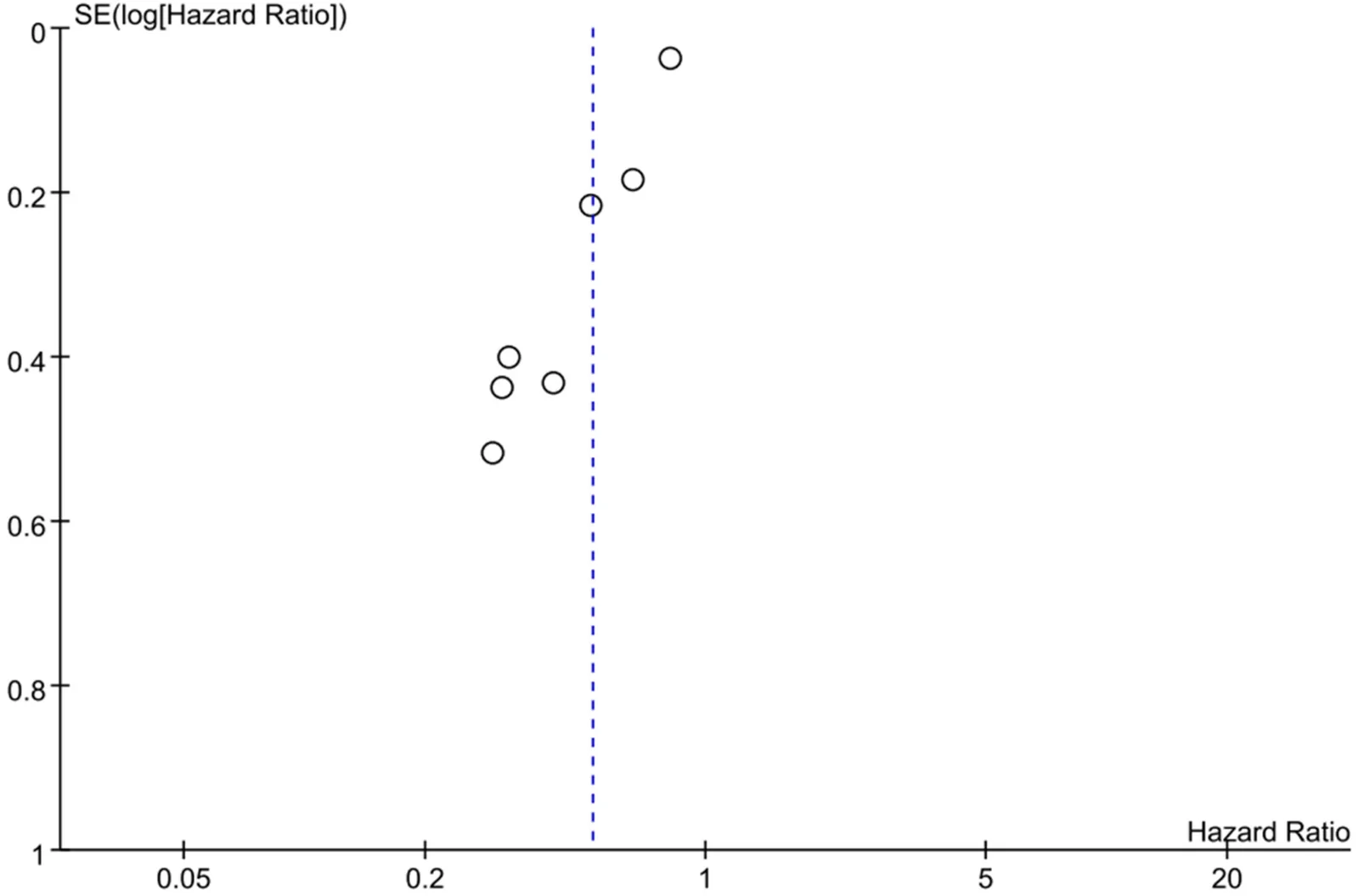
Funnel plot showing publication bias.

Egger's test, Begg's test, and a funnel plot were used to assess the publication bias in this meta-analysis. The funnel plot showed an asymmetric distribution of studies, suggesting potential publication bias (Figure 4). Begg's test (*P* = 0.239) did not detect significant publication bias, whereas Egger's test suggested the presence of publication bias (*P* = 0.003). To further evaluate the potential impact of publication bias, we performed a trim-and-fill analysis. This method estimated two missing studies. After adjusting for publication bias, the pooled HR was 0.68 (95% CI = 0.47–0.99, *P* = 0.046), indicating that the protective effect of GLP-1RAs was attenuated but remained statistically significant.

### 3.7 Certainty of evidence

As the primary evidence came from 6 cohort studies and 1 RCT, the initial certainty was rated as low. It was downgraded by one level for serious risk of bias (two cohort studies were rated as serious risk), by one level for inconsistency (*I*^2^ = 71%), by one level for imprecision (the 95% PI crossed the null), and by one level for publication bias (Egger's test *P* = 0.003) (Supplementary Table S5). The overall certainty of evidence was rated as very low.

## 4 Discussion

In this study, a systematic review and meta-analysis were performed to comprehensively evaluate the association between GLP-1RAs and the risk of AF recurrence after catheter ablation. The results showed that the use of GLP-1RAs significantly reduced the risk of AF recurrence after catheter ablation, indicating that GLP-1RAs may play an important protective role in the secondary prevention of AF recurrence following the procedure.

Current clinical studies on GLP-1RAs and the risk of AF recurrence are relatively limited, and their findings are controversial. Some studies have shown that GLP-1RAs can significantly reduce the risk of AF recurrence after catheter ablation, which is consistent with the findings of our study (43, 44). The study by Li et al. showed a trend toward a reduction in AF recurrence rate in the GLP-1RAs group, although no statistical significance was observed (28), suggesting a potential protective effect of GLP-1RAs on post-ablation AF recurrence. By integrating existing data, this meta-analysis enhanced statistical power and provided more reliable evidence for the protective effect of GLP-1RAs in reducing the risk of AF recurrence after catheter ablation.

Moderate heterogeneity was observed among the included studies. Subgroup analyses consistently demonstrated a protective effect of GLP-1RAs on AF recurrence after catheter ablation, regardless of sample size, study design and follow-up duration. This indicated that heterogeneity did not compromise the results of the meta-analysis, and that the findings are highly robust. The subgroup analysis further showed that the protective effect reported in studies with a sample size of fewer than 200 cases (HR = 0.34) was stronger than that in studies with more than 200 cases (HR = 0.70). This may be related to publication bias in small-sample studies or differences in the characteristics of the included populations. Both retrospective and prospective studies showed consistent protective effects of GLP-1RAs on AF recurrence after catheter ablation, suggesting that study design did not significantly affect the robustness of the results. In the subgroup analysis by follow-up duration, the results for ≤12 months and >12 months were similar, indicating that the protective effect of GLP-1RAs against AF recurrence after catheter ablation is not a transient, short-term effect but rather a sustained, stable long-term benefit. It should be noted that all subgroup analyses were exploratory in nature. Given the limited number of studies included in each subgroup, the observed difference should be interpreted with caution. These findings are hypothesis-generating and warrant confirmation in future studies.

GLP-1RAs are synthetic analogs of the GLP-1 that regulate blood glucose levels via the GLP-1 receptor (45, 46). Current clinical and basic research has revealed that GLP-1RAs may exert anti-arrhythmic effects through multiple mechanisms.

Metabolic regulation and indirect protective effects: GLP-1RAs can reduce the risk of AF indirectly by reducing body weight, regulating blood pressure, lowering blood lipids, and enhancing insulin sensitivity (25, 47–49).

Anti-fibrotic effects and structural remodeling: GLP-1RAs can delay atrial remodeling and reduce atrial fibrosis by inhibiting the TGF-*β* signaling pathway, thereby reducing myofibroblast proliferation and collagen deposition (19).

Inhibition of inflammatory responses: GLP-1RAs can also inhibit NF-*κ*B activation through multiple signaling pathways, effectively attenuating the production of key inflammatory mediators such as TNF-*α*, IL-6, and IL-1*β*, thereby significantly reducing myocardial fibrosis, electrophysiological disturbances, and structural remodeling (25).

Electrophysiological modulation: Studies have shown that GLP-1RAs can reduce the L-type calcium current, sodium-calcium exchange current, and late sodium current in pulmonary vein cardiomyocytes, increase the sarcoplasmic reticulum calcium content of these cells, and reduce the arrhythmogenic effects of the pulmonary veins, thereby exerting anti-arrhythmic potential (50, 51).

Regulation of atrial electrical remodeling: GLP-1RAs can also regulate calmodulin expression to stabilize calcium homeostasis, enhance calcium transients and sarcoplasmic reticulum calcium content, and reduce calcium leakage, thereby preventing the onset and recurrence of atrial fibrillation (52, 53).

Regulation of microbial metabolites: In recent years, research on the gut-heart axis has provided new perspectives for understanding the anti-arrhythmic mechanisms of GLP-1RAs. Accumulating evidence indicates that metabolites produced by gut dysbiosis, including trimethylamine N-oxide (TMAO), lipopolysaccharides (LPS), and short-chain fatty acids (SCFAs), can influence the development and progression of arrhythmias through various mechanisms, such as structural remodeling, electrophysiological remodeling, and inflammatory regulation (54, 55). As a novel class of glucose-lowering agents, GLP-1RAs can reduce serum LPS levels, restore the expression of the intestinal tight junction protein Zonula Occludens-1 (ZO-1), and decrease systemic inflammation caused by LPS translocation, thereby modulating the gut-heart axis (56, 57). Animal experiments have confirmed that semaglutide can reduce TMAO levels in a rat model of heart failure while alleviating cardiac fibrosis and improving diastolic function (58). These findings suggest that GLP-1RAs may reduce AF recurrence risk indirectly by improving intestinal barrier function and reducing LPS/TMAO-mediated inflammatory responses. However, no study has directly confirmed that GLP-1RAs prevent arrhythmias by modulating gut microbiota and their metabolites, which represents an important direction for future research.

Differential effects of various GLP-1RAs on atrial remodeling: Current evidence supports the notion that the anti-arrhythmic effect of GLP-1RAs is a class effect, meaning that different GLP-1 RAs confer similar benefits in reducing AF recurrence risk. The study by Karakasis et al. showed that subgroup analysis by drug type revealed no significant difference in AF risk reduction (*P* = 0.610) (59). This finding suggests that when selecting GLP-1RAs, clinicians may make decisions based more on individual patient factors (e.g., tolerability, BMI) rather than on drug-specific anti-arrhythmic effects.

Although current evidence supports a class effect for GLP-1RAs in reducing AF recurrence, the possibility of differential effects among specific agents cannot be completely ruled out. Tirzepatide, as a dual GIP/GLP-1 agonist, has distinct pharmacological properties that could theoretically translate into different cardiovascular effects. However, the available evidence is insufficient to support this hypothesis. Future comparative studies are warranted to determine whether clinically meaningful differences exist among various GLP-1RAs in terms of AF recurrence prevention.

In summary, GLP-1RAs reduce the risk of AF recurrence through multiple mechanisms, including metabolic regulation and the mitigation of atrial structural and electrical remodeling.

The findings of this study have certain implications for clinical practice. For patients with type 2 diabetes, obesity or metabolic syndrome undergoing catheter ablation, the use of GLP-1 RAs may not only help manage blood glucose and body weight but also provide additional benefits in reducing the risk of AF recurrence. Given that the currently included studies are predominantly observational and a causal relationship has not yet been established, large-scale RCTs are still needed for further verification in the future.

Compared with previously published meta-analyses (43, 44), this study makes several unique contributions. First, we included the most recent studies published up to June 2026 (27, 39, 42), updating the evidence base from 3 to 5 studies to 7 studies and incorporating the first RCT in this field. Second, we systematically searched four Chinese biomedical databases (CNKI, WanFang, VIP, and CBM), which were not searched in previous meta-analyses, thereby reducing the risk of language bias. Third, we performed subgroup analyses stratified by sample size, study design, and follow-up duration with formal interaction tests, which were not conducted in previous meta-analyses. Fourth, we conducted cumulative meta-analysis, prediction interval calculation, and Trim-and-Fill adjustment to assess the robustness of our findings. Fifth, we applied the GRADE framework to assess the certainty of evidence for the primary outcome, which has not been reported in previous meta-analyses. Sixth, we integrated the latest mechanistic insights (2024–2026), including the gut–heart axis and differential effects among GLP-1RAs, which have not been discussed in previous meta-analyses. These contributions are summarized in Supplementary Material (Supplementary Table S6).

Several limitations of our meta-analysis should be acknowledged. First, observational studies predominated in this analysis, making the results susceptible to selection bias and confounding factors. In particular, confounding by indication and healthy-user bias cannot be fully excluded. Patients prescribed GLP-1RAs may have better overall health awareness, more frequent follow-up, or higher adherence to lifestyle modifications compared with non-users, which could lead to an overestimation of the protective effect. Additionally, GLP-1RAs are typically prescribed to patients with obesity or type 2 diabetes, who inherently carry a higher risk of AF recurrence, potentially confounding the observed association. Although all included studies used propensity score matching or multivariable adjustment to control for measured confounders, residual or unmeasured confounding may still exist.

Additionally, two studies (33, 34) reported only binary outcome data, which were converted to HRs using the Watkins (35) method for inclusion in the meta-analysis. To assess the robustness of this conversion, we performed a stratified subgroup sensitivity analysis stratified by the original effect measure type. The HR subgroup and RR subgroup both showed consistent protective directions, supporting the robustness of the primary findings. However, data conversion itself may introduce some degree of imprecision, and the converted HR estimates may be less precise than those derived directly from survival analyses. This limitation should be considered when interpreting our findings.

Third, some of the primary eligible studies did not specify the agent, dosage, or duration of GLP-1RAs therapy, which prevented us from performing dose-response analyses and limited the clinical applicability of our findings. Future studies should systematically report the specific agent used, along with dosing regimens and treatment duration, to enable more detailed comparative analyses and inform optimal clinical decision-making.

Fourth, heterogeneity in ablation techniques (radiofrequency ablation vs. cryoballoon ablation vs. pulsed field ablation) was not considered. These technical differences may influence post-operative AF recurrence rates and may interact with the effect of GLP-1RAs. Due to the lack of stratified data by ablation modality in the original publications, we were unable to perform subgroup analyses to formally evaluate this potential interaction. Different ablation techniques have distinct lesion formation and recurrence characteristics, which could theoretically affect the protective effect of GLP-1RAs. Whether the therapeutic benefits of GLP-1RAs differ by ablation modality remains inconclusive and requires further validation in larger-scale studies.

Fifth, the data of this meta-analysis were derived from aggregated data of published studies. Due to the absence of individual patient-level data (IPD), we were unable to construct time-to-event models, adjust for individual-level confounding factors, perform competing risk analyses, or conduct subgroup analyses based on patient characteristics. Subsequent collaborative IPD meta-analyses will help to provide more robust evidence.

Sixth, the detection methods for AF recurrence after the blanking period varied across studies. These methodological differences may lead to underestimation or overestimation of recurrence rates depending on monitoring intensity, thereby affecting the reliability of the pooled estimates. The lack of standardized detection protocols remains a significant limitation and should be addressed in future studies with uniform monitoring strategies.

Seventh, the 95% prediction interval of our study was 0.24–1.09, which crossed 1, indicating that although the current pooled result is statistically significant, the robustness of the conclusion is limited and future studies may yield different results.

Eighth, small-study effects may have influenced the results. The cumulative meta-analysis ordered by sample size showed that smaller studies produced a larger effect size (HR = 0.27), whereas the pooled HR gradually decreased to 0.51 as larger studies were added. This suggests that small studies may overestimate the protective effect of GLP-1RAs on AF recurrence. This phenomenon is common in meta-analyses, as small studies often report larger effect sizes due to publication bias or methodological limitations. Nevertheless, the cumulative HR after inclusion of all studies remained statistically significant (HR = 0.51, 95% CI = 0.36–0.71), and the 95% confidence interval never crossed 1 throughout the cumulative process. This indicates that although small studies may have amplified the effect size, the direction and statistical significance of our conclusion are not dominated by small studies and are relatively robust.

Ninth, publication bias may affect the results. The asymmetric funnel plot and significant Egger's test (*P* = 0.003) suggested possible publication bias, and the Trim-and-Fill analysis estimated two missing studies, with an adjusted HR of 0.68 (95% CI = 0.47–0.99). Although the adjusted effect remained statistically significant, the upper limit of the CI approached 1. This suggests that unpublished null results may exist and the protective effect of GLP-1RAs may be overestimated. However, the limited number of included studies constrains the statistical power of these publication bias tests. Furthermore, the observed funnel plot asymmetry may partly reflect genuine between-study heterogeneity (*I*^2^ = 71%) rather than publication bias alone. Importantly, a sensitivity analysis using RVE yielded results consistent with the primary analysis, indicating that our conclusions are not driven by model assumptions, small-study bias, or any single dominant study.

Consequently, the overall certainty of evidence was rated as very low. Therefore, the observed protective effect of GLP-1RAs should be interpreted as suggestive rather than definitive, and further high-quality RCTs are warranted to confirm these findings.

## 5 Conclusions

Based on pooled data from seven clinical studies, this meta-analysis provides suggestive evidence that the use of GLP-1RAs may be associated with a reduced risk of AF recurrence after catheter ablation. Sensitivity and subgroup analyses yielded consistent results supporting the robustness of these findings. However, the 95% prediction interval crossed the null, indicating that the protective effect is not definitive. Given the limited number of included studies and the moderate heterogeneity observed, these findings should be interpreted with caution. Large-scale, well-designed RCTs are needed to confirm these findings.

## Data Availability

The raw data supporting the conclusions of this article will be made available by the authors, without undue reservation
