# Supplementary material for: Association of glucagon-like peptide-1 receptor agonists with atrial fibrillation recurrence after catheter ablation: a systematic review and meta-analysis
Source: Front Cardiovasc Med. 2026 Aug 27;13:1868320. doi: 10.3389/fcvm.2026.1868320 (PMC13557988; doi:10.3389/fcvm.2026.1868320)
Supplement: Supplementary file 1 [file Table1.docx]

| **Supplementary Table 1. Search strategies** | | |
| --- | --- | --- |
| Database | Search date | Search strategy |
| PubMed | April 10, 2026 | (((((((((liraglutide[Title/Abstract]) OR (semaglutide[Title/Abstract])) OR (dulaglutide[Title/Abstract])) OR (exenatide[Title/Abstract])) OR (lixisenatide[Title/Abstract])) OR (albiglutide[Title/Abstract])) OR (tirzepatide[Title/Abstract])) OR (((((((((((((((GLP-1 Agonists[Title/Abstract]) OR (Agonists, GLP-1[Title/Abstract])) OR (GLP 1 Agonists[Title/Abstract])) OR (GLP-1 Receptor Agonists[Title/Abstract])) OR (GLP 1 Receptor Agonists[Title/Abstract])) OR (GLP1 Agonists[Title/Abstract])) OR (Agonists, GLP1[Title/Abstract])) OR (GLP-1R Agonists[Title/Abstract])) OR (Agonists, GLP-1R[Title/Abstract])) OR (GLP 1R Agonists[Title/Abstract])) OR (GLP1R Agonists[Title/Abstract])) OR (Agonists, GLP1R[Title/Abstract])) OR (Incretin Mimetics[Title/Abstract])) OR (GLP-1 Analogs[Title/Abstract])) OR (GLP 1 Analogs[Title/Abstract]))) OR ("Glucagon-Like Peptide-1 Receptor Agonists"[Mesh])) AND ((((((((((((((((((((((((((Atrial Fibrillations[Title/Abstract]) OR (Fibrillation, Atrial[Title/Abstract])) OR (Fibrillations, Atrial[Title/Abstract])) OR (Auricular Fibrillation[Title/Abstract])) OR (Auricular Fibrillations[Title/Abstract])) OR (Fibrillation, Auricular[Title/Abstract])) OR (Fibrillations, Auricular[Title/Abstract])) OR (Persistent Atrial Fibrillation[Title/Abstract])) OR (Atrial Fibrillation, Persistent[Title/Abstract])) OR (Atrial Fibrillations, Persistent[Title/Abstract])) OR (Fibrillation, Persistent Atrial[Title/Abstract])) OR (Fibrillations, Persistent Atrial[Title/Abstract])) OR (Persistent Atrial Fibrillations[Title/Abstract])) OR (Familial Atrial Fibrillation[Title/Abstract])) OR (Atrial Fibrillation, Familial[Title/Abstract])) OR (Atrial Fibrillations, Familial[Title/Abstract])) OR (Familial Atrial Fibrillations[Title/Abstract])) OR (Fibrillation, Familial Atrial[Title/Abstract])) OR (Fibrillations, Familial Atrial[Title/Abstract])) OR (Paroxysmal Atrial Fibrillation[Title/Abstract])) OR (Atrial Fibrillation, Paroxysmal[Title/Abstract])) OR (Atrial Fibrillations, Paroxysmal[Title/Abstract])) OR (Fibrillation, Paroxysmal Atrial[Title/Abstract])) OR (Fibrillations, Paroxysmal Atrial[Title/Abstract])) OR (Paroxysmal Atrial Fibrillations[Title/Abstract])) OR ("Atrial Fibrillation"[Mesh])) |
| Embase | April 10, 2026 | ('glp 1 agonist':ti,ab OR 'glp 1 receptor agonist':ti,ab OR 'glucagon like peptide 1 agonist':ti,ab OR 'glucagon like peptide 1 receptor stimulating agent':ti,ab OR 'glucagon-like peptide-1 receptor agonists':ti,ab OR 'incretin mimetics':ti,ab OR 'long acting glp 1 agonist':ti,ab OR 'long acting glp 1 receptor agonist':ti,ab OR 'long acting glucagon like peptide 1 agonist':ti,ab OR 'long acting glucagon like peptide 1 receptor agonist':ti,ab OR 'glucagon like peptide 1 receptor agonist':ti,ab OR 'semaglutide':ti,ab OR 'liraglutide':ti,ab OR 'tirzepatide':ti,ab OR 'albiglutide':ti,ab OR 'lixisenatide':ti,ab OR 'exendin 4':ti,ab OR 'dulaglutide':ti,ab OR 'glucagon like peptide 1 receptor agonist'/exp) AND ('atrial fibrillation':ti,ab OR 'auricular fibrillation':ti,ab OR 'AF':ti,ab OR 'A-fib':ti,ab OR 'atrial fibrillation'/exp) |
| Web of Science | April 10, 2026 | "GLP-1 Analogs" OR "glucagon-like peptide-1 receptor agonist*" OR "GLP-1 agonist*" OR "GLP-1 receptor agonist*" OR "GLP-1R agonist*" OR "incretin mimetic*" OR "semaglutide" OR "liraglutide" OR "dulaglutide" OR "exenatide" OR "lixisenatide" OR "tirzepatide" (Topics) and "Atrial Fibrillation" OR "auricular fibrillation" OR "atrium fibrillation" OR "AF" OR "A-fib" OR "AFib" OR "heart atrial fibrillation" OR "cardiac atrial fibrillation" (Topic) |
| Cochrane Library | April 10, 2026 | ((MeSH descriptor: [Glucagon-Like Peptide-1 Receptor Agonists] explode all trees) OR ((liraglutide):ti,ab,kw OR (semaglutide):ti,ab,kw OR (dulaglutide):ti,ab,kw OR (exenatide):ti,ab,kw OR (lixisenatide):ti,ab,kw OR (albiglutide):ti,ab,kw OR (tirzepatide):ti,ab,kw)) AND ((MeSH descriptor: [Atrial Fibrillation] explode all trees) OR (((atrial):ti,ab,kw OR (atria):ti,ab,kw OR (atrium):ti,ab,kw) AND (fibrillat*):ti,ab,kw)) |
| VIP | April 10, 2026 | SU=('胰高血糖素样肽-1受体激动剂' OR 'GLP-1' OR '胰高血糖素样肽-1' OR 'GLP-1受体激动剂' OR '司美格鲁肽' OR '利拉鲁肽' OR '度拉糖肽' OR '艾塞那肽' OR '利西拉肽' OR '替尔泊肽') AND SU=('心房颤动' OR '房颤' OR '心房纤颤') |
| CNKI | April 10, 2026 | SU=('胰高血糖素样肽-1受体激动剂' OR 'GLP-1' + '胰高血糖素样肽-1' OR 'GLP-1受体激动剂' OR '司美格鲁肽' OR '利拉鲁肽' OR '度拉糖肽' OR '艾塞那肽' OR '利西拉肽' OR '替尔泊肽') AND SU=('心房颤动' OR '房颤' OR '心房纤颤') |
| Wanfang Data | April 10, 2026 | 主题:(胰高血糖素样肽\-1受体激动剂 OR GLP\-1 OR 胰高血糖素样肽\-1 OR GLP\-1受体激动剂 OR 司美格鲁肽 OR 利拉鲁肽 OR 度拉糖肽 OR 艾塞那肽 OR 利西拉肽 OR 替尔泊肽) and 主题:(心房颤动 OR 房颤 OR心房纤颤) |
| CBM | April 10, 2026 | (("心房颤动"/EXP) OR ("房颤" OR "心房纤颤")) AND (("胰高血糖素样肽-1受体激动剂"/EXP) OR "GLP-1受体激动剂" OR "GLP-1激动剂" OR "肠促胰素模拟物" OR "艾塞那肽" OR "利拉鲁肽" OR "度拉糖肽" OR "司美格鲁肽" OR "替西帕肽") |
