# Supplementary material for: Association of glucagon-like peptide-1 receptor agonists with atrial fibrillation recurrence after catheter ablation: a systematic review and meta-analysis
Source: Front Cardiovasc Med. 2026 Aug 27;13:1868320. doi: 10.3389/fcvm.2026.1868320 (PMC13557988; doi:10.3389/fcvm.2026.1868320)
Supplement: Supplementary file 2 [file Table2.docx]

| **Supplementary Table S2a. Original proportions of included studies** | | | |
| --- | --- | --- | --- |
| Study | Participants  (GLP-1RAs / Control) | Freedom from AF recurrence  (GLP-1RAs / Control) | *P*-value |
| Zou et al. (2024) | 61 / 62 | 88.5% vs 67.7% | 0.026 |
| Salerno et al. (2025) | 53 / 53 | 90.6% vs 71.7% | 0.013 |
| Abbreviations: AF, atrial fibrillation | | |  |

| **Supplementary Table S2b. Conversion calculations using Watkins et al. method** | | | | | |
| --- | --- | --- | --- | --- | --- |
| Study | p (GLP-1RAs) | p (Control) | ln(p_G) | ln(p_C) | HR |
| Zou et al. (2024) | 0.885 | 0.677 | -0.1222 | -0.3901 | 0.313 |
| Salerno et al. (2025) | 0.906 | 0.717 | -0.0987 | -0.3327 | 0.297 |
| Abbreviations: p = proportion free from AF recurrence; HR = ln(p_G) / ln(p_C); HR, hazard ratio. | | | | | |
