# Supplementary material for: Association of glucagon-like peptide-1 receptor agonists with atrial fibrillation recurrence after catheter ablation: a systematic review and meta-analysis
Source: Front Cardiovasc Med. 2026 Aug 27;13:1868320. doi: 10.3389/fcvm.2026.1868320 (PMC13557988; doi:10.3389/fcvm.2026.1868320)
Supplement: Supplementary file 3 [file Table3.docx]

| **Supplementary Table S3. Numerical weights for manual validation of pooled HR** | | | |
| --- | --- | --- | --- |
| Study | HR (95% CI) | Weight (%) | Numerical weight |
| Ciconte et al. 2026 | 0.52 (0.34-0.80) | 18.1% | 6.37 |
| Goldberger et al. 2026 | 0.32 (0.15-0.71) | 10.4% | 3.68 |
| Guo et al. 2025 | 0.66 (0.46-0.95) | 19.7% | 6.93 |
| Salerno et al. 2025 | 0.30 (0.11-0.82) | 7.5% | 2.64 |
| Tabaja et al. 2024 | 0.42 (0.18-0.98) | 9.6% | 3.37 |
| Venier et al. 2026 | 0.82 (0.76-0.88) | 25.4% | 8.98 |
| Zou et al. 2024 | 0.31 (0.13-0.74) | 9.4% | 3.30 |
| Total | 0.52 (0.37-0.73) | 100% | 35.27 |
| Abbreviations: Numerical weight calculated as *w_i_* = 1/(SE_i_^2^+τ^2^), where τ^2^ = 0.11; HR, hazard ratio | | | |
