# Supplementary material for: Association of glucagon-like peptide-1 receptor agonists with atrial fibrillation recurrence after catheter ablation: a systematic review and meta-analysis
Source: Front Cardiovasc Med. 2026 Aug 27;13:1868320. doi: 10.3389/fcvm.2026.1868320 (PMC13557988; doi:10.3389/fcvm.2026.1868320)
Supplement: Supplementary file 4 [file Table4.docx]

| **Supplementary Table S4a. RR for AF recurrence in studies with binary outcome data** | | | | | |
| --- | --- | --- | --- | --- | --- |
| Studies | p (GLP-1RAs) | p (Control) | RR | lnRR | 95% CI |
| Zou et al. 2024 | 11.5% | 32.3% | 0.36 | -1.022 | 0.16-0.78 |
| Salerno et al. 2025 | 9.4% | 28.3% | 0.33 | -1.104 | 0.13-0.85 |
| Abbreviations: AF, atrial fibrillation; p = proportion of AF recurrence; RR = p(GLP-1RAs) / p (Control);  RR, risk ratio; GLP-1 RAs, glucagon-like peptide-1 receptor agonists; CI, confidence interval. | | | | | |

| **Supplementary Table S4b. Sensitivity analysis comparing HR and RR subgroup estimates** | | | |
| --- | --- | --- | --- |
| Subgroup | Pooled effect size | 95% CI | Direction |
| HR subgroup (5 studies) | 0.60 | 0.44-0.82 | Protective |
| RR subgroup (2 studies) | 0.35 | 0.19-0.64 | Protective |
| Primary analysis (7 studies) | 0.52 | 0.37-0.73 | Protective |
| Abbreviations: HR, hazard ratio; RR, risk ratio; CI, confidence interval. | | | |
