# Supplementary material for: Association of glucagon-like peptide-1 receptor agonists with atrial fibrillation recurrence after catheter ablation: a systematic review and meta-analysis
Source: Front Cardiovasc Med. 2026 Aug 27;13:1868320. doi: 10.3389/fcvm.2026.1868320 (PMC13557988; doi:10.3389/fcvm.2026.1868320)
Supplement: Supplementary file 5 [file Table5.docx]

| **Supplementary Table S5. GRADE assessment for the primary outcome** | | |
| --- | --- | --- |
| Domain | Grade | Explanation |
| Starting level | Low | One RCT could provide high-level evidence, but its contribution is limited due to high risk of bias.  six cohort studies started as “low” certainty |
| Risk of bias | -1 | One RCT: high risk;  two cohort studies: serious risk;  four cohort studies: moderate risk |
| Inconsistency | -1 | Moderate heterogeneity: I² = 71% |
| Indirectness | 0 | Population, intervention, comparator, and outcomes directly aligned with the PICO question |
| Imprecision | -1 | 95% PI crossed the null: 95% PI = 0.24-1.09 |
| Publication bias | -1 | Egger’s test significant, *P* = 0.003 |
| Abbreviations: GRADE, Grading of Recommendations Assessment, Development and Evaluation;  RCT, Randomized controlled trial; PI, Prediction interval | | |
