# Supplementary material for: Association of glucagon-like peptide-1 receptor agonists with atrial fibrillation recurrence after catheter ablation: a systematic review and meta-analysis
Source: Front Cardiovasc Med. 2026 Aug 27;13:1868320. doi: 10.3389/fcvm.2026.1868320 (PMC13557988; doi:10.3389/fcvm.2026.1868320)
Supplement: Supplementary file 6 [file Table6.docx]

| **Supplementary Table S6. Comparison of the current meta-analysis with previous meta-analyses** | | | |
| --- | --- | --- | --- |
| Dimension | Karakasis et al. (2024) | Kilic et al. (2026) | Our study (2026) |
| Search cutoff date | June, 2024 | September, 2025 | June, 2026 |
| Number of included studies | 3 | 5 | 7 |
| RCT included | No | No | Yes |
| Chinese databases searched | No | No | Yes |
| Subgroup analyses | No | No | Yes |
| Cumulative meta-analysis | No | No | Yes |
| Prediction interval | No | No | Yes |
| Trim-and-Fill adjustment | No | No | Yes |
| GRADE certainty assessment | No | No | Yes |
| Latest mechanistic insights | No | No | Yes |
| Abbreviations: GRADE, Grading of Recommendations Assessment, Development and Evaluation | | | |
